# Activation of Cannabinoid Receptor 2 Ameliorates DSS-Induced Colitis through Inhibiting NLRP3 Inflammasome in Macrophages

**DOI:** 10.1371/journal.pone.0155076

**Published:** 2016-09-09

**Authors:** Ping Ke, Bo-Zong Shao, Zhe-Qi Xu, Wei Wei, Bin-Ze Han, Xiong-Wen Chen, Ding-Feng Su, Chong Liu

**Affiliations:** 1 Department of Pharmacology, School of Pharmacy, Second Military Medical University, Shanghai, China; 2 Institute of Quality and Standard for Agro-products, Zhejiang Academy of Agricultural Sciences; Hangzhou, Zhejiang, China; 3 Cardiovascular Research Center, Temple University School of Medicine, Philadelphia, Pennsylvania, 19140, United States of America; Virginia Polytechnic Institute and State University, UNITED STATES

## Abstract

Activation of cannabinoid receptor 2 (CB2R) ameliorates inflammation, but the underlying mechanism remains unclear. In the present study, we examined whether activation of CB2R could suppress the nucleotide-binding domain and leucine-rich repeat protein 3 (NLRP3) inflammasome. In peritoneal macrophages isolated from C57BL/6 mice, LPS/DSS challenge for 24 h increased the expression of the components of NLRP3 inflammasome NLRP3, Casp-1 p20/Casp-1 p45 ratio, proIL-1β and IL-1β and also enhanced autophagy (LC3-II/LC3-I ratio, Beclin-1 and SQSTM1). Pretreatment of peritoneal macrophages with HU 308, a selective CB2R agonist, attenuated LPS/DSS-induced NLRP3 inflammasome activation, but further enhanced autophagy. In comparison with wild-type (WT) control, peritoneal macrophages from CB2R knockout (KO) mice had more robust NLRP3 inflammasome activation and attenuated autophagy upon LPS/DSS challenge. Knockdown autophagy-related gene 5 (*Atg5*) with a siRNA in peritoneal macrophages attenuated the inhibitory effects of HU 308 on LPS/DSS-induced NLRP3 inflammasome activation *in vitro*. *In vivo*, HU308 treatment attenuated DSS-induced colitis mice associated with reduced colon inflammation and inhibited NLRP3 inflammasome activation in wild-type mice. In CB2R KO mice, DSS-induced inflammation and NLRP3 inflammasome activation were more pronounced than those in WT control. Finally, we demonstrated that AMPK-mTOR-P70S6K signaling pathway was involved in this CB2R-mediated process. We conclude that activation of CB2R ameliorates DSS-induced colitis through enhancing autophagy that may inhibit NLRP3 inflammasome activation in macrophages.

## Introduction

Cannabinoid receptor 2 (CB2R) belongs to the family of G-protein-coupled receptors (GPCR), with seven transmembrane α-helices, a glycosylated extracellular amino-terminus and an intracellular carboxyl-terminus [1,2]. Unlike cannabinoid receptor 1 (CB1R) which is majorly located in central nervous system, CB2R is mainly expressed in the periphery, especially in the immune system including macrophage and other immune cells, acting as immune and inflammatory modulator [3,4]. Established evidence has indicated that activating CB2R plays a protective role in inflammation- and autoimmune-related diseases [5–7], but the underlying mechanism remains unclear.

The inflammasome has emerged as a key player in innate immunity and inflammation [8,9]. Upon ligand sensing, inflammasome components assemble and self-oligomerize, leading to caspase-1 (Casp-1) activation and maturation of proIL-1β and proIL-18 into bioactive cytokines, namely IL-1β and IL-18. Both IL-1β and IL-18 then play a pivotal role in the initiation and amplification of the inflammatory process [10]. Among several types of inflammasomes, the nucleotide-binding domain and leucine-rich repeat protein 3 (NLRP3) inflammasome is the best characterized subtype. Activation of NLRP3 inflammasome mainly includes two steps: the first, or the priming step, is the activation of NF-κB and inflammasome components including NLRP3 and proIL-1β to induce NLRP3 inflammasome initiation, and the secondary step is the activation of NLRP3 inflammasome assembly and inflammatory reaction [11]. It has been reported that abnormal activation of NLRP3 inflammasome is related to several inflammation- and autoimmune-related diseases [12–14].

Here we investigated the role of CB2R in NLRP3 inflammasome activation in macrophages and explored its relevance to dextran sulphate sodium (DSS)-induced experimental colitis. Our results demonstrate for the first time that activating CB2R could suppress the initiation and activation of NLRP3 inflammasome by enhancing autophagy in peritoneal macrophages challenged with LPS/DSS, which contributes to the protective effect of CB2R on DSS-induced experimental colitis.

## Materials and Methods

### Animal care and use

C57BL/6 mice (8–10 weeks old, male) were purchased from Shanghai Super-B&K Laboratory Animal Corp., Ltd. (Shanghai, China). CB2R knockout (KO) mice on C57BL/6 background (8–10 weeks old, male) were purchased from Jackson Laboratory (Bar Harbor, MA) (B6.129P2-Cnr2^*tm1Dgen*^/J, Stock Number: 005786) and kept at 22°C under a 12-h light/dark cycle with unlimited access to water and standard rodent diet. All experiments were approved and conducted in accordance with the guidelines of the Animal Care Committee of Second Military Medical University.

### Mice peritoneal macrophages culture and treatment

Mice peritoneal macrophages were collected as described previously [15–17]. In brief, CB2R KO mice and age- and sex-matched WT controls were injected 10% thioglycollate (i.p.) for 3 days. Resident peritoneal macrophages were collected from the peritoneal cavity by flushing with 5 mL of ice-cold Hanks’ balanced salt solution containing 10 U/mL heparin. Macrophages were plated at a density of 5×10^4^/mL in Dulbecco’s modified essential medium supplemented with 10% fetal calf serum, and were left to adhere for 4 hours in a humidified atmosphere at 37°C with 5% CO_2_. Then cells were washed twice, and the remaining cells were primed with 10 ng/ml lipopolysaccharides (LPS, Sigma, Louis, MO, USA) for 1 h, and then were stimulated with 3% DSS (mol. wt. 36,000 to 50,000 kDa, MP Biomedicals LLC, Santa Ana, CA, USA) in the presence or absence of 10 μM HU 308 (TOCRIS Bioscience, Bristol, BS, UK) for 24 h [18,19]. In another set of experiments, peritoneal macrophages from WT and CB2R KO mice were stimulated with/without LPS/DSS for 24 h.

### Transient transfection and siRNA

The following siRNAs against *Atg5* (Gene ID: 11793) were synthesized by Genepharm Biotech (Shanghai, China): siRNA1, 5’-CUCUCUAUCAGGAUGAUTT-3’, 5’-AUCUCAUCCUGAUAGAGAGTT-3’; siRNA2, 5’-GACGUUGGUAACUGACAAATT-3’, 5’-UUUGUCAGUUACCAACGUCTT-3’; siRNA3, 5’-GCGGUUGAGGCUCACUUUATT-3’, 5’-UAAAGUGAGCCUCAACCGCTT-3’; siRNA4, 5’-GCUACCCAGAUAACUUUCUTT-3’, 5’-AGAAAGUUAUCUGGGUAGCTT-3’. All siRNAs consisted of 21 nucleotides and contained symmetric 3’ overhangs of two deoxythymidines. Mice peritoneal macrophages were transfected with siRNAs as previously reported [20,21].

### Immunoblot analysis

Proteins were extracted from the tissue or cultured cells using a standard extraction reagent supplemented with the protease inhibitor (KANGCHEN; Shanghai, China). The protein concentration was determined using a BCA protein assay kit (Beyotime Institute of Biotechnology; Haimen, China). The proteins were separated using SDS PAGE and electro-transferred to nitrocellulose membranes as described previously [22,23], and incubated with a primary antibody for 8–12 h at 4°C. Samples were then incubated with an IRDye800CW-conjugated secondary antibody (Rockland; Gilbertsville, PA, USA) for 1h at 25°C. The image was acquired with the Odyssey infrared imaging system (Li-Cor Bioscience; Lincoln, NE, USA). All immunoblotting experiments were repeated for at least 3 times.

### Quantitative real-time polymerase chain reaction (QT-PCR)

The relative mRNA expression of IL-1β was determined by quantitative real-time polymerase chain reaction using the ABI PRISM 7700 Sequence Detection System based on published methods with optimal concentrations of primers and probes. Mouse IL-1β primer (forward: 5’-CTCGTGCTGTCGGACCCCAT-3’ and reverse, 5’- AGTGTTCGTCTCGTGTTCGGAC -3’) was added at a final concentration of 900 nM. For internal controls, mouse GAPDH primers (forward: 5’-GTATGACTCCACTCACGGCAAA-3’ and reverse, 5’-GGTCTCGCTCCTGGAAGATG-3’**)** were added at final concentrations of 500 nM, respectively. The thermal cycler conditions were 35 cycles of 94°C for 20 seconds, 55°C for 20 seconds, and 72°C for 30 seconds. Data were analyzed using Sequence Detection System software version 1.9.1. All samples were run in duplicate.

### Enzyme-linked immunosorbent assay (ELISA)

The level of IL-1β, IL-6 and TNF-α in cell supernatants were quantified using commercial ELISA kits (R&D system, New York, NY, USA).

### Immunofluorescence staining and fluorescence microscopy

RAW 264.7 cells (murine macrophage from blood) were incubated overnight at 37°C on glass coverslips, followed by the treatments mentioned above. Cells were fixed in 200 μl of 4% paraformaldehyde for 15 min, washed with phosphate-buffered saline (PBS) before blocking with 5% bovine serum albumin in PBST, and incubated with primary antibodies overnight at 4°C. Double immunofluorescent staining was completed by Alexa-488 or Alexa-647-labeled secondary antibody (Invitrogen, USA) incubation for 1 h at room temperature. After being washed, slides were mounted with Vectashield mounting medium containing DAPI (Vector Laboratories, Burlingame, CA, USA) and colocalization was observed using a confocal laser scanning microscope (Fluoview FV1000; Olympus, Tokyo, Japan). Image Pro Plus 6.0 software (Media Cybernetics, Bethesda, MD, USA) was employed to analyze colocalization, expressed as the Pearson correlation coefficient as previously reported [24,25].

### Induction of colitis

Colitis was induced in C57BL/6 wild type (WT) and CB2R KO mice with 3% DSS dissolved in drinking water given *ad libitum* (days 1–8) as previously described [26,27]. Body weight and bloody stool were monitored once a day until day 8. Changes of body weight are indicated as loss of baseline body weight as a percentage. Then mice were anesthetized with phenobarbital sodium (60 mg/kg, i.p.) and euthanized by cervical dislocation. Postmortem, the colon was removed and pieces of colonic tissue were used for *ex vivo* analysis. The presence of occult or gross blood per rectum, and stool consistency were determined by two investigators blinded to the treatment groups. A scoring system was used to assess diarrhea and the presence of occult or overt blood in the stool [28,29]. For histology, rings of the transverse part of the colon were fixed in 4% buffered formalin and embedded in paraffin. Sections were stained with H&E according to standard protocols. Histological scoring was performed in a blinded way by a pathologist. Focally increased numbers of inflammatory cells in the lamina propria were scored as 1, confluence of inflammatory cells extending into the submucosa as 2 and transmural extension of the infiltrate as 3. For tissue damage, discrete lymphoepithelial lesions were scored as 1, mucosal erosions as 2, and extensive mucosal damage and/or extension through deeper structures of the bowel wall as 3. The two equally weighted subscores (cell infiltration and tissue damage) were added and the combined histological colitis severity score ranged from 0 to 6. During the experimental procedure, euthanasia is performed prior to the experimental endpoint if there were rectal prolapse, loss of over 15% body weight, or signs of pain and distress such as poor grooming, decreased activity, and hunched posture.

### Experimental protocols

#### Experiment 1: Effects of activating CB2R on NLRP3 inflammosome initiation and activation in peritoneal macrophages challenged with LPS/DSS

Peritoneal macrophages isolated from C57BL/6 mice were primed with 10 ng/ml LPS for 1 h, and then stimulated with 3% DSS in the presence or absence of 10 μM HU 308 for 24 h. In another set of experiments, peritoneal macrophages from WT and CB2R KO mice were stimulated with or without LPS/DSS for 24 h. The expression of NLRP3, Casp-1 p20/Casp-1 p45 ratio and proIL-1β were analyzed using Western blot. The expression of IL-1β mRNA was tested by QT-PCR. IL-1β, IL-6 and TNF-α in the supernatant were measured by ELISA.

#### Experiment 2: Effects of activating CB2R on autophagy in peritoneal macrophages challenged with LPS/DSS

Peritoneal macrophages were isolated and primed with 10 ng/ml LPS for 1 h, and then were stimulated with 3% DSS in the presence or absence of 10 μM HU 308 for 24 h. In another set of experiments, peritoneal macrophages from WT and CB2R KO mice were stimulated with or without LPS/DSS for 24 h. Cell lysate were collected and LC3-II/LC3-I ratio, Beclin-1 and SQSTM1 were analyzed using Western blot.

#### Experiment 3: Influence of autophagy on inhibitory effect of CB2R on NLRP3 inflammasome initiation and activation in peritoneal macrophages challenged with LPS/DSS

After transfection with control siRNA or *Atg5* siRNA, RAW 264.7 cells were primed with 10 ng/ml LPS for 1 h, and then were stimulated with 3% DSS in the presence or absence of 10 μM HU 308 for 24 h. Cells were fixed and immunostaining was performed as described above. Colocalization of NLRP3 with ASC and NLRP3 with Casp-1 was assessed with a confocal laser scanning microscope. In another set of experiments, after transfection with control siRNA or *Atg5* siRNA, RAW246.7 cells were primed with LPS (10 ng/ml) for 1 h, and then were stimulated with 3% DSS in the presence or absence of HU 308 (10 μM) for 24 h. The expression of Casp-1 p20/Casp-1 p45 ratio was analyzed using Western blot. IL-1βsecretion in the supernatant was measured by ELISA.

#### Experiment 4: Effects of activating CB2R on symptoms and severity of DSS-induced colitis in mice

C57BL/6 mice were treated with vehicle or HU 308 (1 mg/kg, i.p.) once a day from day 0 to day 8 when receiving 3% DSS, the control mice received tap water. In another set of experiments, WT and CB2R KO mice received 3% DSS for 8 days, the control mice were given tap water. Changes of body weight, bloody stool score, colon length and histologic score were examined as mentioned above.

#### Experiment 5: Effects of activating CB2R on NLRP3 inflammosome activation and autophagy in colon from DSS-induced colitis mice

C57BL/6 mice were treated with vehicle or HU 308 (1 mg/kg, i.p.) once a day from day 0 to day 8 when drinking 3% DSS, the control animals received tap water. In another set of experiments, WT and CB2R KO mice received 3% DSS for 8 days, the control mice were given tap water. Colon tissues were isolated from mice at day 8, and then the expression of NLRP3, Casp-1 p20/Casp-1 p45 ratio, proIL-1β, LC3-II/LC3-I, Beclin-1 and SQSTM1 were determined by Western blot.

#### Experiment 6: Influence of autophagy on the protective effect of CB2R activation and on the inhibitory effect of CB2R on NLRP3 inflammasome activation in DSS-induced colitis

C57BL/6 mice were received 3% DSS, DSS + HU 308 (1 mg/kg, i.p.) or DSS + HU 308 (1 mg/kg, i.p.) + 3-methyladenine (3-MA, an autophagy inhibitor, 10 mg/kg, i.p.) daily for 8 days. The control mice were given tap water. Body weight, bloody stool score, colon length and histologic score were examined. Colon tissues were isolated from mice at day 8, and then the expression of Casp-1 p20/Casp-1 p45 ratio, proIL-1β was examined by Western blot.

#### Experiment 7: The signaling cascades in the regulation of autophagy by CB2R activation in peritoneal macrophages challenged with LPS/DSS

Peritoneal macrophages from WT and CB2R KO mice were isolated and treated with/without LPS/DSS for 24 h. Colon tissues were isolated from mice at day 8 after DSS-induced colitis. The phosphorylation of AMP-activated protein kinase (p-AMPK/AMPK), mammalian target of rapamycin (p-mTOR/mTOR) and p70 ribosomal protein S6 Kinase (p-P70S6K/P70S6K) in peritoneal macrophages and colon tissues were analyzed using Western blot. In another set of experiments, peritoneal macrophages from C57BL/6 mice were challenged by LPS/DSS+ vehicle, LPS/DSS + HU 308, LPS/DSS + compound C (10 μM, an AMPK inhibitor, Sigma, Louis, MO, USA) and LPS/DSS + compound C + HU 308. The expression of NLRP3, Casp-1 p20/Casp-1 p45 ratio, proIL-1β was analyzed using Western blot.

### Statistical analysis

Data are expressed as means ± SEM. For nonparametric data, a Kruskal-Wallis test followed by a Dunn’s post-test was used. For continuous variables, the statistical differences between groups were determined by one-way analysis of variance followed by a Student-Newman-Keuls test. Statistical analyses were performed using GraphPad Prism 4 software (GraphPad Software, San Diego, CA, USA). P < 0.05 was considered statistically significant.

## Results

### CB2R activation suppresses NLRP3 inflammasome activation in peritoneal macrophages challenged with LPS/DSS

**Fig 1:**
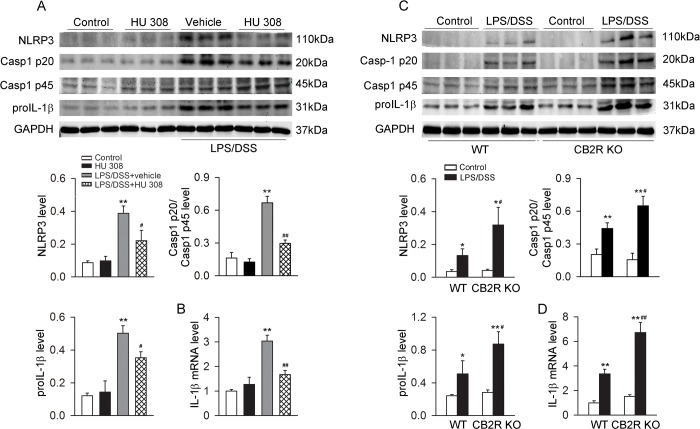
CB2R negatively regulates NLRP3 inflammasome activation in peritoneal macrophages stimulated with LPS/DSS. After lipopolysaccharides (LPS, 10 ng/ml) priming for 1 h, peritoneal macrophages were stimulated with 3% dextran sulphate sodium (DSS) in the presence or absence of HU 308 (10 μM) for 24 h. In another set of experiments, peritoneal macrophages from wild-type (WT) mice and cannabinoid receptor 2 (CB2R) knockout (KO) mice were isolated and stimulated with/without LPS/DSS for 24 h. The expression of NLRP3, Casp-1 p20/Casp-1 p45 ratio, proIL-1β and IL-1β mRNA were analyzed. (A and B) Pre-incubated with HU 308 significantly decreased the expressions of NLRP3, Casp-1 p20/Casp-1 p45 ratio, proIL-1β and IL-1β mRNA. n = 6 per group. **P<0.01 *vs*. control, #P<0.05 *vs*. LPS/DSS*+*vehicle; ##P<0.01 *vs*. LPS/DSS+vehicle. (C and D) The expressions of NLRP3, Casp-1 p20/Casp-1 p45 ratio, proIL-1β and IL-1β mRNA were significantly increased in CB2R KO group. n = 6 per group. *P<0.05 *vs*. control, **P<0.01 *vs*. control, #P<0.05 *vs*. WT, ##P<0.01 *vs*. WT.

**Fig 2:**
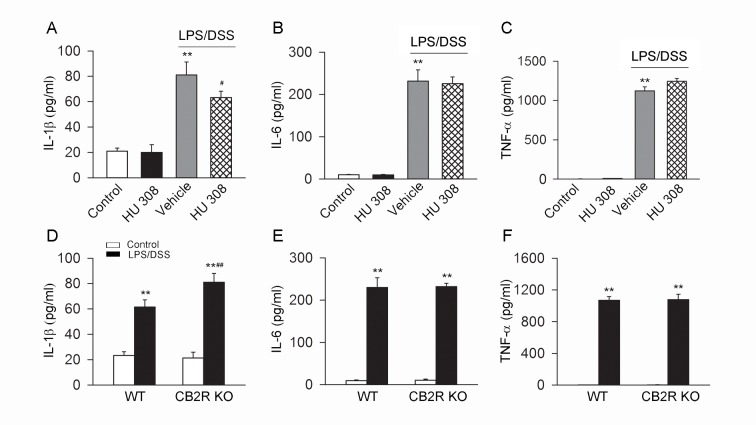
CB2R inhibits the production of IL-1β but not IL-6 and TNF-α in cultured peritoneal macrophages stimulated with LPS/DSS. After LPS (10 ng/ml) priming for 1 h, peritoneal macrophages were stimulated with 3% DSS in the presence or absence of HU 308 (10 μM) for 24 h. In another set of experiments, peritoneal macrophages from WT mice and CB2R KO mice were isolated and stimulated with/without LPS/DSS for 24 h. The levels of IL-1β, IL-6 and TNF-α were measured by ELISA. (A-C) Pre-incubated with HU 308 significantly decreased the level of IL-1β but have no effect on the levels of IL-6 and TNF-α. n = 6 per group. **P<0.01 *vs*. control, #P<0.05 *vs*. LPS/DSS+vehicle. (D-F) In comparison to WT group, the level of IL-1β was significantly increased in CB2R KO group, while the levels of IL-6 and TNF-α weren’t increased. n = 6 per group. **P<0.01 *vs*. control, ##P<0.01 *vs*. WT.

LPS/DSS stimulation increased the protein abundance of NLRP3, Casp-1 p20/Casp-1 p45 ratio, proIL-1β and the mRNA of IL-1β in C57BL/6 mice peritoneal macrophages. Pre-incubation with HU 308 significantly attenuated the increase of protein abundance of NLRP3, Casp-1 p20/Casp-1 p45 ratio, proIL-1β and IL-1β mRNA in macrophages induced by LPS/DSS (Fig 1A and 1B). After LPS/DSS challenge, the protein abundance of NLRP3, Casp-1 p20/Casp-1 p45 ratio, proIL-1β and the IL-1β mRNA were increased significantly more in CB2R KO than in WT peritoneal macrophages (Fig 1C and 1D). Moreover, LPS/DSS stimulation increased IL-1β, IL-6 and TNF-α in supernatant. Pre-incubation with HU 308 significantly decreased IL-1β but not IL-6 and TNF-α in the supernatant of the cultured C57BL/6 mice peritoneal macrophages challenged with LPS/DSS (Fig 2A–2C). In comparison with WT peritoneal macrophages, CB2R KO ones secreted more IL-1β but unchanged IL-6 and TNF-α upon LPS/DSS stimulation (Fig 2D–2F).

### CB2R activation increases autophagy in peritoneal macrophages challenged with LPS/DSS

**Fig 3:**
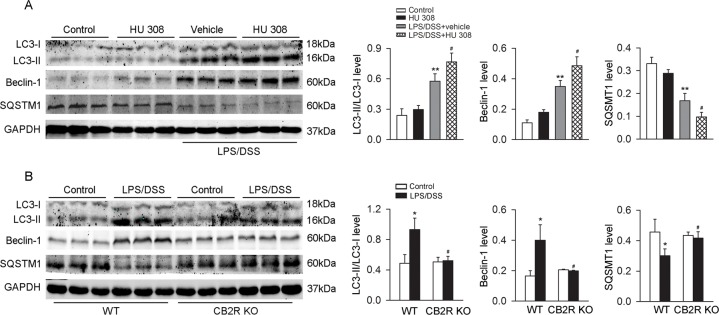
CB2R augments autophagy in peritoneal macrophages stimulated with LPS/DSS. After LPS (10 ng/ml) priming for 1 h, peritoneal macrophages were stimulated with 3% DSS in the presence or absence of HU 308 (10 μM) for 24 h. In another set of experiments, peritoneal macrophages from WT and CB2R KO mice were isolated and stimulated with/without LPS/DSS for 24h. Macrophages were collected and LC3-II/LC3-I, Beclin-1 and SQSTM1 were analyzed using Western blot. (A) Pre-incubated with HU 308 significantly increased the level of LC3-II/LC3-I, Beclin-1 and decreased the level of SQSTM1. n = 6 per group. **P<0.01 *vs*. control, #P<0.05 *vs*. vehicle. (B) The expression of LC3-II/LC3-I and Beclin-1 were decreased and the expression of SQSTM1 was increased in CB2R KO group. n = 6 per group. *P<0.05 *vs*. control, #P<0.05 *vs*. WT.

Upon LPS/DSS stimulation, LC3-II/LC3-I ratio and Beclin-1 abundance were increased and SQSTM1 (a selective substrate of autophagy) abundance was decreased in C57BL/6 mice peritoneal macrophages. Pre-incubation with HU 308 further enhanced the effects of LPS/DSS on the LC3-II/LC3-I ratio, Beclin-1 and SQSTM1 (Fig 3A). When compared with WT peritoneal macrophages, LC3-II/LC3-I ratio and Beclin-1 abundance were decreased and SQSTM1 abundance was increased in CB2R KO groups upon LPS/DSS challenge (Fig 3B).

### Inducing autophagy mediates the inhibition of CB2R on NLRP3 inflammasome initiation and activation in RAW 264.7 cells challenged with LPS/DSS

**Fig 4:**
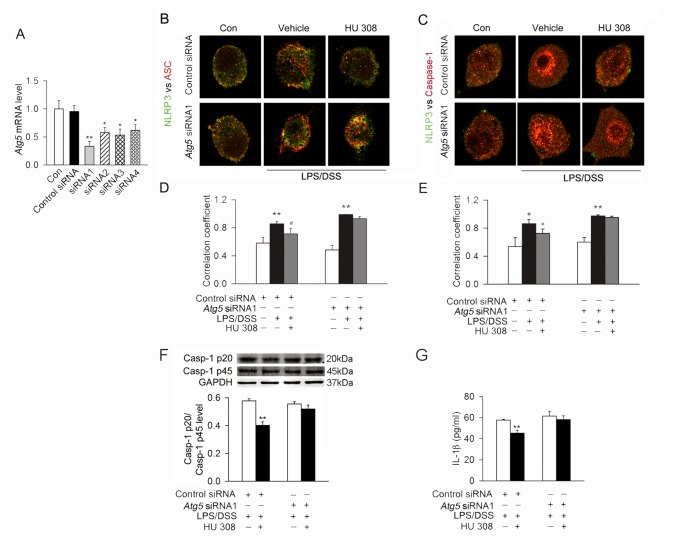
Autophagy mediates the inhibitory effect of CB2R on NLRP3 inflammasome initiation and activation stimulated with LPS/DSS *in vitro*. (A) The quantitative expression of mRNA in RAW264.7 cells after transfection with control siRNA or *Atg5* siRNAs. n = 6 per group. *P<0.05 *vs*. the control siRNA, **P<0.01 *vs*. the control siRNA. (B and C) After transfection with control siRNA or *Atg5* siRNA, RAW264.7 cells were primed with LPS (10 ng/ml) for 1 h, and then were stimulated with 3% DSS in the presence or absence of HU 308 (10 μM) for 24 h. Cells were fixed and colocalization of NLRP3 with ASC and NLRP3 with Casp-1 were observed using a confocal laser scanning microscope. Representative images of the colocalization of NLRP3 (green) with ASC or Casp-1 (red). (D and E) Quantitative analysis showing the fold change in Pearson coefficient correlation (PCC) for the colocalization of NLRP3 with ASC and NLRP3 with Casp-1. *Atg5* siRNA attenuated the inhibitory effect of HU 308 the colocalization of NLRP3 with ASC and NLRP3 with Casp-1. n = 6 per group. *P<0.05 *vs*. control, **P<0.01 *vs*. control, #P<0.05 *vs*. LPS/DSS. (F and G) After transfection with control siRNA or *Atg5* siRNA, RAW246.7 cells were primed with LPS (10 ng/ml) for 1 h, and then were stimulated with 3% DSS in the presence or absence of HU 308 (10 μM) for 24 h. Western blot was conducted to test Casp-1 p20 in supernatants and Casp-1 p45 and GAPDH in lysates, and the expression of Casp-1 p20/Casp-1 p45 ratio was analyzed. IL-1β secretion in the supernatant was tested by ELISA. HU 308 decreased the level of Casp-1 p20/Casp-1 p45 ratio and IL-1β secretion. *Atg5* siRNA attenuated the inhibitory effect of HU 308 on Casp-1 p20/Casp-1 p45 ratio and IL-1β secretion. n = 8 per group. **P<0.01 *vs*. LPS/DSS.

LPS/DSS stimulation induced the colocalization of NLRP3 with apoptosis-associated speck-like protein containing a CARD (ASC) and NLRP3 with Casp-1 in RAW264.7 cells, which was partially prevented by pre-incubation with HU 308. Blocking autophagy by *Atg5* siRNA attenuated the inhibitory effect of HU 308 on the colocalization of NLRP3 with ASC and NLRP3 with Casp-1 (Fig 4A–4E). Moreover, HU 308 also decreased the Casp-1 p20/Casp-1 p45 ratio and IL-1β secretion in RAW264.7 cells stimulated with LPS/DSS. This effect of HU 308 was attenuated by *Atg5* siRNA (Fig 4F and 4G).

### CB2R activation alleviates but CB2R KO aggravates the symptoms and colon inflammation of DSS-induced colitis mice

**Fig 5:**
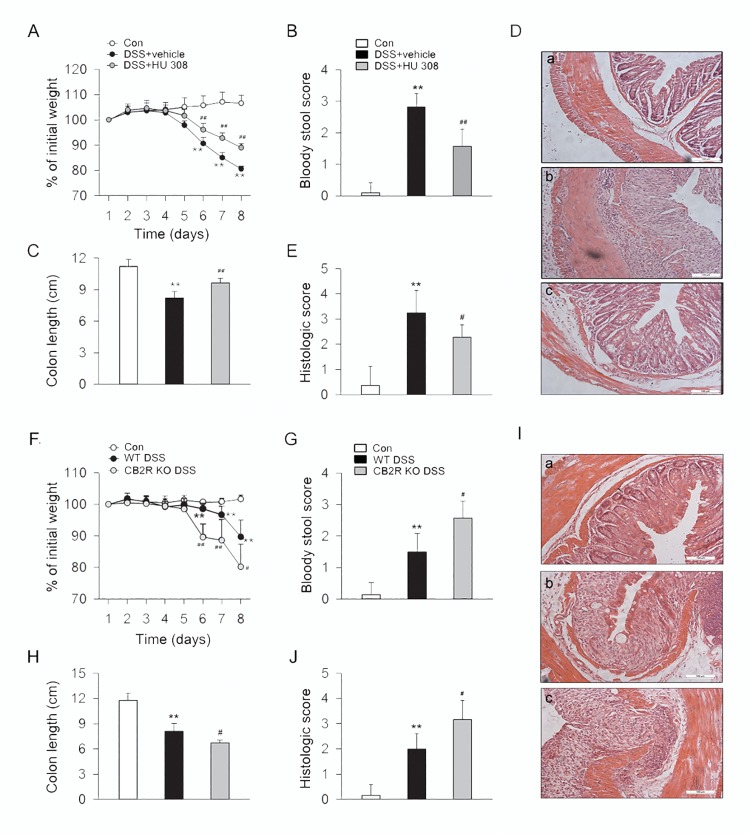
CB2R activation ameliorates the symptoms and colon inflammation in DSS-induced colitis mice. C57BL/6 mice were treated with vehicle or HU 308 (1 mg/kg, i.p.) once a day from day 0 to day 8 while receiving 3% DSS, the control mice were given tap water. In another set of experiments, WT and CB2R KO mice received 3% DSS for 8 days, the control mice were given tap water. Body weight, the presence of occult or gross blood per rectum, and stool consistency were determined by two investigators blinded to the treatment groups. Postmortem, the colon was removed and pieces of colonic tissue were used for *ex vivo* analysis. Treatment of HU 308 significantly reduced body weight loss (A), bloody stool score (B), increment of colon length (C) and alleviated colon inflammation (D-E). n = 7 per group. **P<0.01 *vs*. the control group; #P<0.05 *vs*. the DSS + vehicle group, ##P<0.01 *vs*. the DSS + vehicle group. Compared with WT group, body weight loss (F), bloody stool score (G), reduction of colon length (H) and colon inflammation (I-J) were significantly increased in CB2R KO group. n = 10 per group. **P<0.01 *vs*. the control group; #P<0.05 *vs*. the WT DSS group, ##P<0.01 *vs*. the WT DSS group.

After drinking 3% DSS water for 8 days, mice developed a severe illness characterized by the presence of sustained weight loss, bloody diarrhea, and severe colon inflammation associated with hyperemia, ulceration and bowel wall thickening leading to an increase of colon length reduction. During this process, one of 10 mice in CB2R KO group died of exacerbated colonic inflammation and exited experiment prior to the experimental endpoint. Our results showed that treatment with HU 308 (1 mg/kg, i.p.) significantly improved symptoms of weight loss, bloody diarrhea, colon length, and colon inflammation, with alleviated inflammatory infiltration in mucosa and submucosa in DSS-induced colitis mice (Fig 5A–5E). In comparison to WT mice, DSS induced a more severe illness and aggravated inflammatory infiltration in colon wall in CB2R KO mice (Fig 5F–5J).

### CB2R activation inhibits NLRP3 inflammasome activation and enhances autophagy in colon from DSS-induced colitis mice

**Fig 6:**
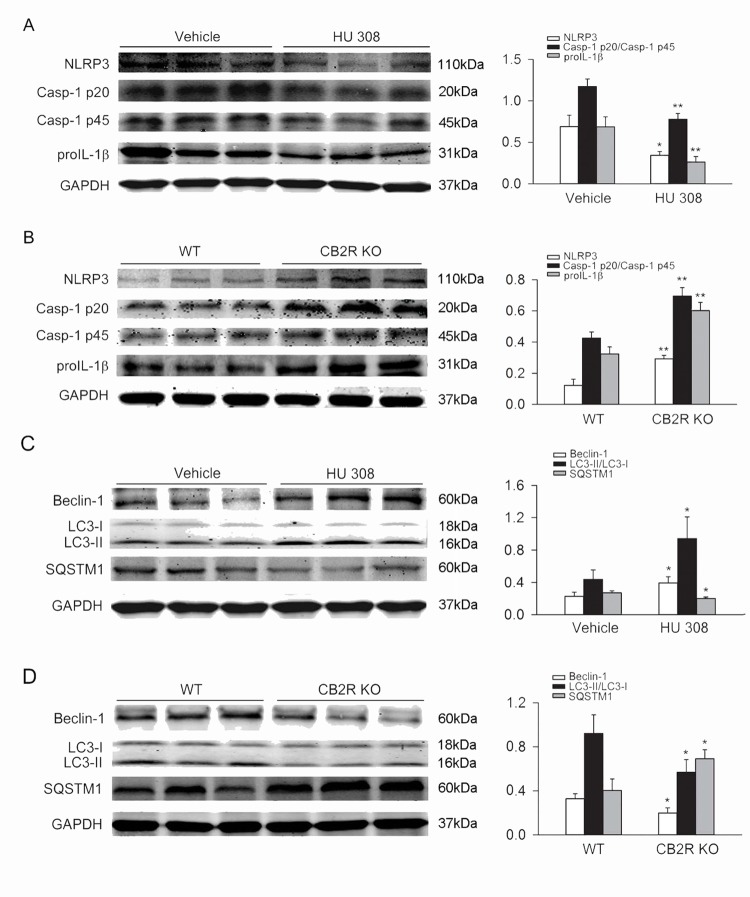
CB2R inhibits NLRP3 inflammasome activation and augments autophagy in colon from DSS-induced colitis mice. C57BL/6 mice were treated with vehicle or HU 308 (1 mg/kg, i.p.) once a day from day 0 to day 8 while receiving 3% DSS, the control animals received saline. In another set of experiments, WT and CB2R KO mice received 3% DSS for 8 days, the control mice were given tap water. Colon tissues were isolated from mice at day 8 and the expression of NLRP3, Casp-1 p20/Casp-1 p45 ratio, proIL-1β, LC3-II/LC3-I, Beclin-1 and SQSTM1 were analyzed by Western blot. (A) HU 308 significantly decreased the expression of NLRP3, Casp-1 p20/Casp-1 p45 ratio and proIL-1β. n = 6 per group. *P<0.05 *vs*. vehicle, **P<0.01 *vs*. vehicle. (B) In comparison with WT mice, the expression of NLRP3, Casp-1 p20/Casp-1 p45 ratio and proIL-1β were significantly increased in CB2R KO group. n = 6 per group. **P<0.01 *vs*. WT. (C) HU 308 significantly increased the expression of LC3-II/LC3-I, Beclin-1 and decreased the expression of SQSTM1. n = 6 per group. *P<0.05 *vs*. vehicle. (D) Compared with WT group, the expression of LC3-II/LC3-I and Beclin-1 was decreased and the expression of SQSTM1 was increased in CB2R KO group. n = 6 per group. *P<0.05 *vs*. WT.

Compared with the vehicle group, treatment with HU 308 (1 mg/kg, i.p.) significantly decreased NLRP3, the Casp-1 p20/Casp-1 p45 ratio and proIL-1β in colon from DSS-induced colitis mice. When compared with WT mice, DSS induced a further increase of NLRP3, Casp-1 p20/Casp-1 p45 ratio and proIL-1β in colon from CB2R KO mice (Fig 6A and 6B). In addition, treatment with HU 308 also significantly increased the LC3-II/LC3-I ratio and Beclin-1 and decreased SQSTM1 in colon from DSS-induced colitis mice. In comparison with WT mice, DSS induced decrease of LC3-II/LC3-I ratio and Beclin-1 and an increase of SQSTM1 in colon from CB2R KO mice (Fig 6C and 6D).

### Blockade of autophagy attenuates the protective effect of CB2R activation on DSS-induced colitis

**Fig 7:**
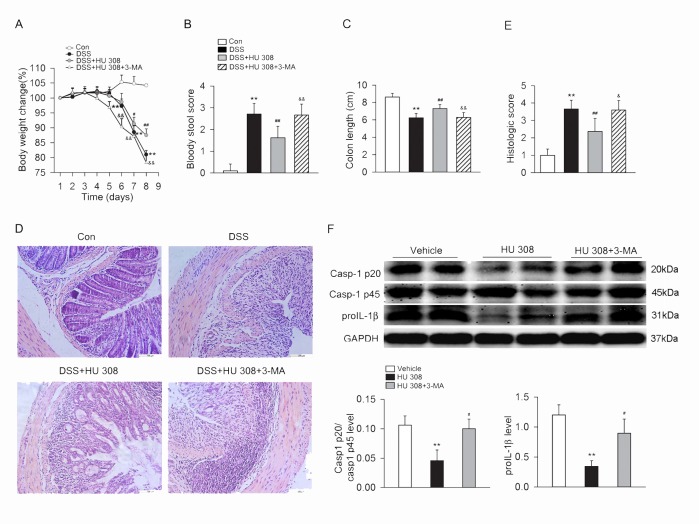
3-MA attenuates the protective effect of HU 308 on DSS-induced colitis in mice and the inhibitory of HU 308 on NLRP3 inflammasome activation in colon from DSS-induced colitis mice. C57BL/6 mice were treated with 3% DSS, DSS + HU 308 (1 mg/kg, i.p.) or DSS + HU 308 (1 mg/kg, i.p.) + 3-MA (10 mg/kg, i.p.) daily for 8 days. The control mice were given tap water. 3-MA significantly inhibited the beneficial effect of HU 308 on body weight loss (A), bloody stool score (B), colon length (C) and colon inflammation (D-E). n = 10 per group. **P<0.01 *vs*. the control group; #P<0.05 *vs*. the DSS group, ##P<0.01 *vs*. the DSS group; &P<0.05 *vs*. the DSS+HU 308 group, &&P<0.01 *vs*. the DSS+HU 308 group. (F) 3-MA also attenuated the inhibitory effect of HU 308 on Casp-1 p20/Casp-1 p45 ratio and proIL-1β in colon. n = 6 per group. **P<0.01 *vs*. vehicle; #P<0.05 *vs*. HU 308 group.

In DSS-induced colitis mice, treatment with 3-MA (10mg/kg, i.p.) attenuated the beneficial effects of HU 308 on weight loss, bloody diarrhea, colon length and colon inflammation, with relatively aggravated inflammatory infiltration in mucosa and submucosa in DSS-induced colitis mice (Fig 7A–7E). Furthermore, 3-MA also attenuated the inhibitory effect of HU 308 on Casp-1 p20/Casp-1 p45 ratio and proIL-1β in colon from DSS-induced colitis mice (Fig 7F).

### CB2R activation increases p-AMPK and decreases p-mTOR, p-P70S6K both *in vitro* and *in vivo*

**Fig 8:**
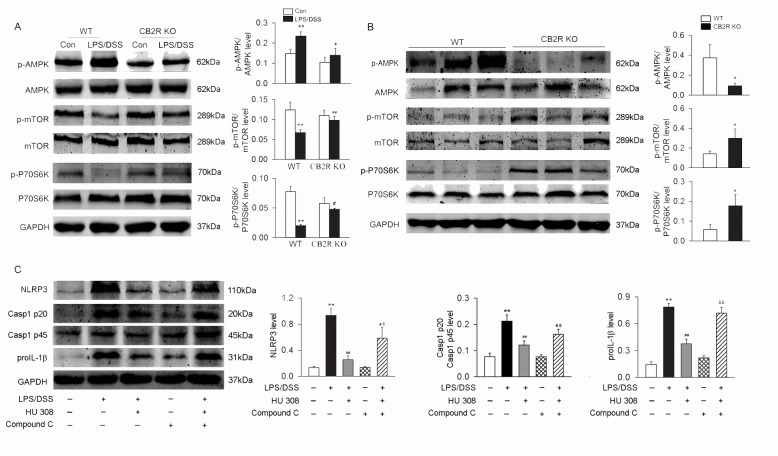
AMPK-mTOR-P70S6K signaling cascades are involved in the regulation of CB2R on autophagy both *in intro* and *in vivo*. Peritoneal macrophages from WT and CB2R KO mice were isolated and primed with LPS (10 ng/ml) for 1 h, and then were stimulated with 3% DSS. Colon tissues were isolated from mice at day 8 after DSS-induced colitis. The expression of p-AMPK/AMPK, p-mTOR/mTOR and p-P70S6K/P70S6K in macrophages and colon tissues were analyzed using Western blot. (A) In colon from DSS-induced colitis mice, the level of p-AMPK was decreased and the levels of p-mTOR and p-P70S6K were increased in CB2R KO mice when compared with those from WT mice. n = 6 per group. *P<0.05 *vs*. control. (B) Compared to WT macrophages, the level of p-AMPK was decreased and the level of p-mTOR and p-P70S6K were increased in CB2R KO group after LPS/DSS stimulation. n = 6 per group. **P<0.01 *vs*. control; #P<0.05 *vs*. WT, ##P<0.01 *vs*. WT. (C) In peritoneal macrophages isolated from C57BL/6 mice, HU 308 decreased the levels of NLRP3, Casp-1 p20/Casp-1 p45 ratio and proIL-1β induced by LPS/DSS. Compound C, an inhibitor of AMPK, partly blocked the inhibitory effects of HU 308 on NLRP3, Casp-1 p20/Casp-1 p45 ratio and proIL-1β.

In colon from DSS-induced colitis mice, the level of p-AMPK was decreased and the level of p-mTOR and p-P70S6K were increased in CB2R KO group when compared with those in WT group (Fig 8A). In WT peritoneal macrophages treated with LPS/DSS, HU 308 stimulation increased the level of p-AMPK and decreased the level of p-mTOR and p-P70S6K. However, in CB2R KO peritoneal macrophages challenged with LPS/DSS, HU 308 stimulation did not affect the levels of p-AMPK, p-mTOR and p-P70S6K (Fig 8B). Furthermore, pre-treatment of the peritoneal macrophage with Compound C (an inhibitor of AMPK) before LPS/DSS stimulation and HU 308 incubation partly blocked the inhibitory effects of HU 308 on the expression of NLRP3, proIL-1β and Casp-1 p20/Casp-1 p45 ratio (Fig 8C).

## Discussion

CB2R has been reported to play an important role in regulating the inflammation process in many inflammation- and autoimmune-related diseases [5–7,30]. Our previous study indicated that activating CB2R could alleviate the symptoms and severity of EAE mice through inhibiting NLRP3 inflammasome activation [31]. Ginhoux et al [32] reported that mouse microglia has different origination compared with macrophages resident in peripheral issues because microglia derive from primitive myeloid progenitors that arise before embryonic day 8 in extra-embryonic yolk sac while other macrophage populations are derived from postnatal hematopoietic progenitors. Therefore, it is quite interesting and inspiring to discuss the role of activating CB2R in NLRP3 inflammasome activation in macrophages in the periphery. Here, in the present work, we found that activating CB2R could not only decrease the protein expression of NLRP3, proIL-1β and Casp-1 p20/Casp-1 p45 ratio but also decrease IL-1β in the supernatant in macrophages stimulated with LPS/DSS, suggesting that activating CB2R suppresses both initiation and activation of the NLRP3 inflammasome. Similar results were observed in colon tissue from DSS-induced colitis mice, where we observed the downregulation or upregulation of NLRP3-related proteins along with activating or inhibiting CB2R respectively. These data indicate that activating CB2R may function as a suppressor of the NLRP3 inflammasome, which also explains the protective effect of CB2R on colitis.

In the past few years, the role of autophagy in the control of inflammation has been extensively investigated. There is increasing evidence that autophagy process contributes greatly to the suppression of NLRP3 inflammasome. Cho et al [33] showed that autophagy contributes to the clearance of extracellular β-amyloid (Aβ) fibrils and suppression of Aβ-induced NLRP3 inflammasome. It has also been reported that autophagy exerts similar effect on inhibition of NLRP3 inflammasome in other cells and disease models, thus playing an important role in inflammatory regulation [34,35]. Consistently, our results demonstrated that autophagy contributed to CB2R-mediated inhibition of NLRP3 inflammasome initiation and activation in peritoneal macrophages stimulated with LPS/DSS as well as in a mouse model of DSS-induced colitis. Nevertheless, it should be noted that there are also some works demonstrating harmful effects of autophagy in colitis. For example, Cheluvappa et al [36] identified suppression of autophagy genes after appendicitis followed by appendectomy in murine models through microarray analysis and gene set enrichment analysis along with the alleviation of experimental colitis, and pointed out that autophagy suppression by appendectomy might lead to less antigen processing and less cross-reactive immunity between microbes and self-antigen in colon, which subsequently resulted in the amelioration of colitis. We consider that the differences in focal points of autophagy may cause this inconsistency. It has been uncovered that autophagy helps promote the antigen-presenting function in antigen presenting cells, especially dendritic cells in inflammatory responses [37], which leads to the enhancement of inflammatory infiltration in colon. However, here we focus on the effects of autophagy on macrophages and its anti-inflammatory functions associated with inhibition of NLRP3 inflammasome. Hence, to ultimately take advantage of the role of autophagy in inflammatory regulation in the treatment of colitis or other inflammation-related diseases, further work is warranted.

Several works have been reported to describe the regulation of CB2R on the autophagy process [38–40]. Louvet et al [38] uncovered an autophagy-dependent pathway of protecting against alcoholic liver disease induced by activating CB2R in liver Kuffer cells. In addition, Casarejos et al [40] showed a neuroprotective role of activating CB2R, which is closely associated with the regulation of inflammatory responses and autophagic process in neurons. Here in our study, we observed that activation of CB2R could significantly promote autophagy process in macrophages, which in turn led to the inhibition of NLRP3 inflammasome activation in mice peritoneal macrophages and experimental colitis mice. Conversely, knockout of CB2R eliminated the protective effects. In addition, blocking autophagy by *Atg5* siRNA in mouse peritoneal macrophages attenuated the effect of CB2R activation on NLRP3 inflammasome. Those were subsequently approved in experimental colitis mice through intraperitoneal injection of 3-MA to block autophagic process. Taken together, these data suggest that inducing autophagy at least partly contributed to CB2R-mediated suppression of NLRP3 inflammasome activation in mice peritoneal macrophages as well as colon of experimental colitis mice.

Dando et al [41] found out that activating CB2R by cannabinoids could induce AMPK-dependent autophagy, which effectively inhibit energetic metabolism and cell proliferation in pancreatic cancer cells. Liu-Bryan et al [42] demonstrated that inducing autophagy through AMPK and SIRT1 pathway provided “stop signals” for oxidative stress, inflammatory responses and matrix catabolic processes in chondrocytes in osteoarthritis. Consistent with these reports, our data demonstrate that CB2R KO decreases the phosphorylation of AMPK, in combination of the increased phosphorylation of mTOR and P70S6K, which leads to the upregulation of autophagy in mouse peritoneal macrophages.

**Fig 9:**
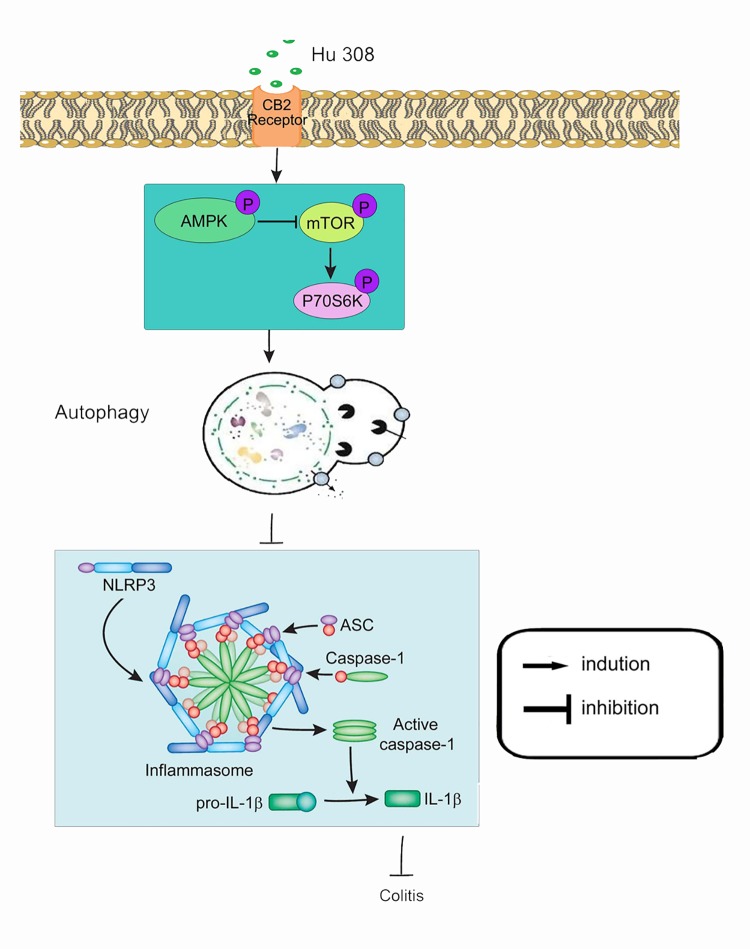
Schematic illustration of the mechanism of CB2R activation inhibits NLRP3 inflammasome in peritoneal macrophages. Once HU 308 binds to CB2R receptor in macrophages, phosphorylates the AMPK-mTOR-P70S6K signaling cascades and thus promotes peritoneal macrophages autophagy. Augment of autophagy inhibits NLRP3 inflammasome initiation and activation, which decreases Casp-1 activation and IL-1β mature and alleviates an inflammatory cascade.

Taken together, we demonstrate that activating CB2R suppresses NLRP3 inflammasome initiation and activation, which is at least partly relying on the induction of autophagy (Fig 9). These findings may be helpful in designing novel therapeutic strategies for colitis. However, it needs to be pointed out that so far, there is still no evidence proving ameliorative effects of CB2R agonist on ulcerative colitis in patients.

## Supporting Information

S1 Fig Peritoneal macrophages were primed with LPS (10ng/ml) for 1 h followed by 3% DSS in the presence or absence of HU 308 (10 μM) for 24 h. In another set of experiments, peritoneal macrophages from WT mice and CB2R KO mice were isolated and stimulated with/without LPS/DSS for 24 h. The mRNA of TNF-α and IL-6 were measured by QT-PCR. **P<0.01 *vs*. control.
(TIF)

S2 Fig Peritoneal macrophages from WT and CB2R KO mice were primed with LPS (10ng/ml) for 1 h followed by the stimulation of ATP (1 mM) for 24 h. The level of IL-1β in supernatant was measured by ELISA. **P<0.01 *vs*. control; ##P<0.01 *vs*. WT.
(TIF)

## Abbreviations

CB2R: cannabinoid receptor 2
NLRP3: leucine-rich repeat protein 3
LPS: lipopolysaccharides
KO: knockout
WT: wild-type
Atg5: autophagy-related gene 5
Casp-1: caspase-1
GPCR: G-protein-coupled receptors
CB1R: cannabinoid receptor 1
DSS: dextran sulphate sodium
IP: thioglycollate
ELISA: enzyme-linked immunosorbent assay
PBS: phosphate-buffered saline
AMPK: AMP-activated protein kinase
mTOR: mammalian target of rapamycin
P70S6K: p70 ribosomal protein S6 kinase
3-MA: 3-methyladenine
ASC: apoptosis-associated speck-like protein containing a CARD
