# Supplementary figures and images for: Activation of Cannabinoid Receptor 2 Ameliorates DSS-Induced Colitis through Inhibiting NLRP3 Inflammasome in Macrophages
Source: PLoS One. 2016 Sep 9;11(9):e0155076. doi: 10.1371/journal.pone.0155076 (PMC5017608; doi:10.1371/journal.pone.0155076)

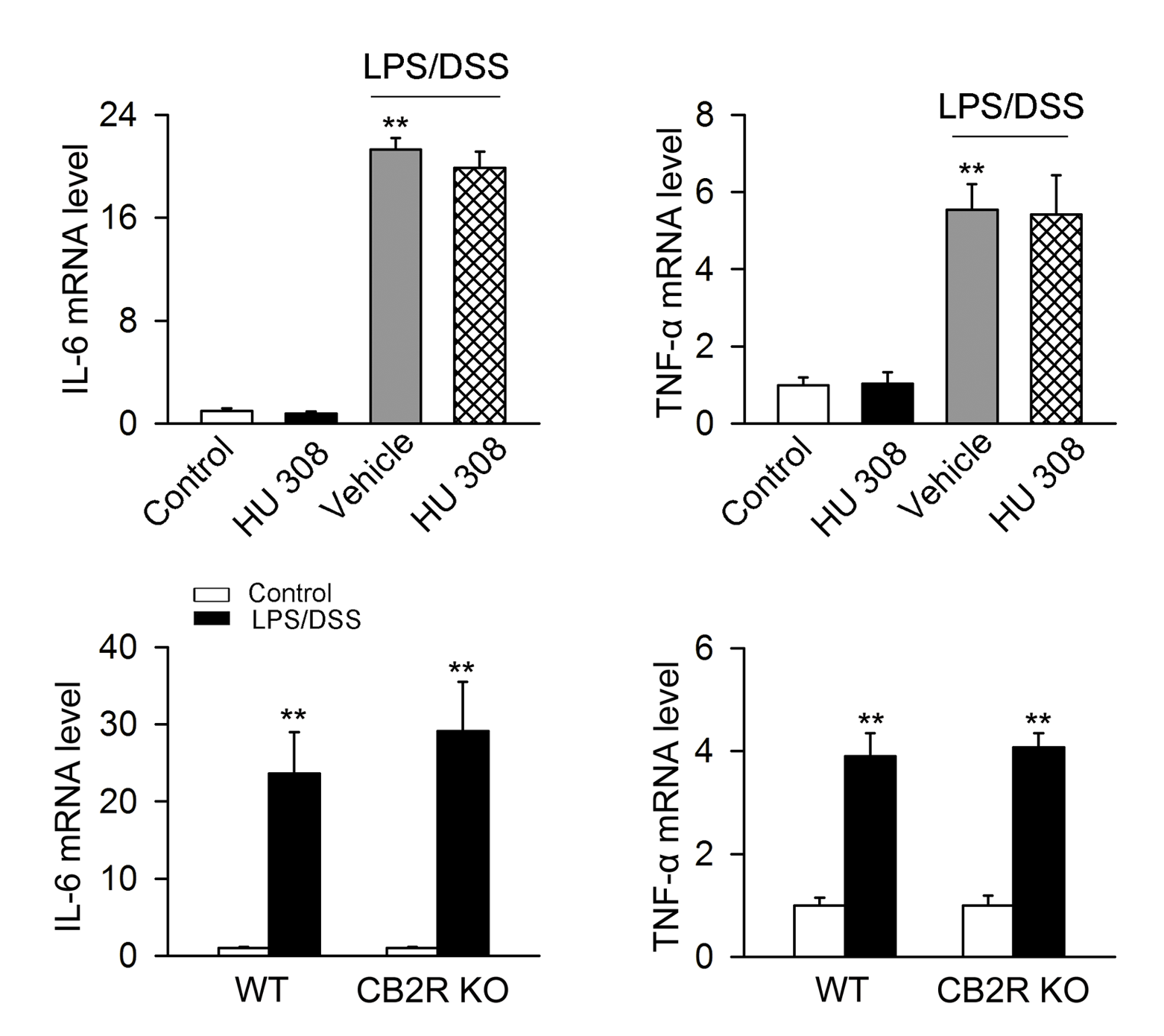

Supplement: S1 Fig — In another set of experiments, peritoneal macrophages from WT mice and CB2R KO mice were isolated and stimulated with/without LPS/DSS for 24 h. The mRNA of TNF-α and IL-6 were measured by QT-PCR. **P<0.01 vs. control. (TIF) [file pone.0155076.s001.tif]

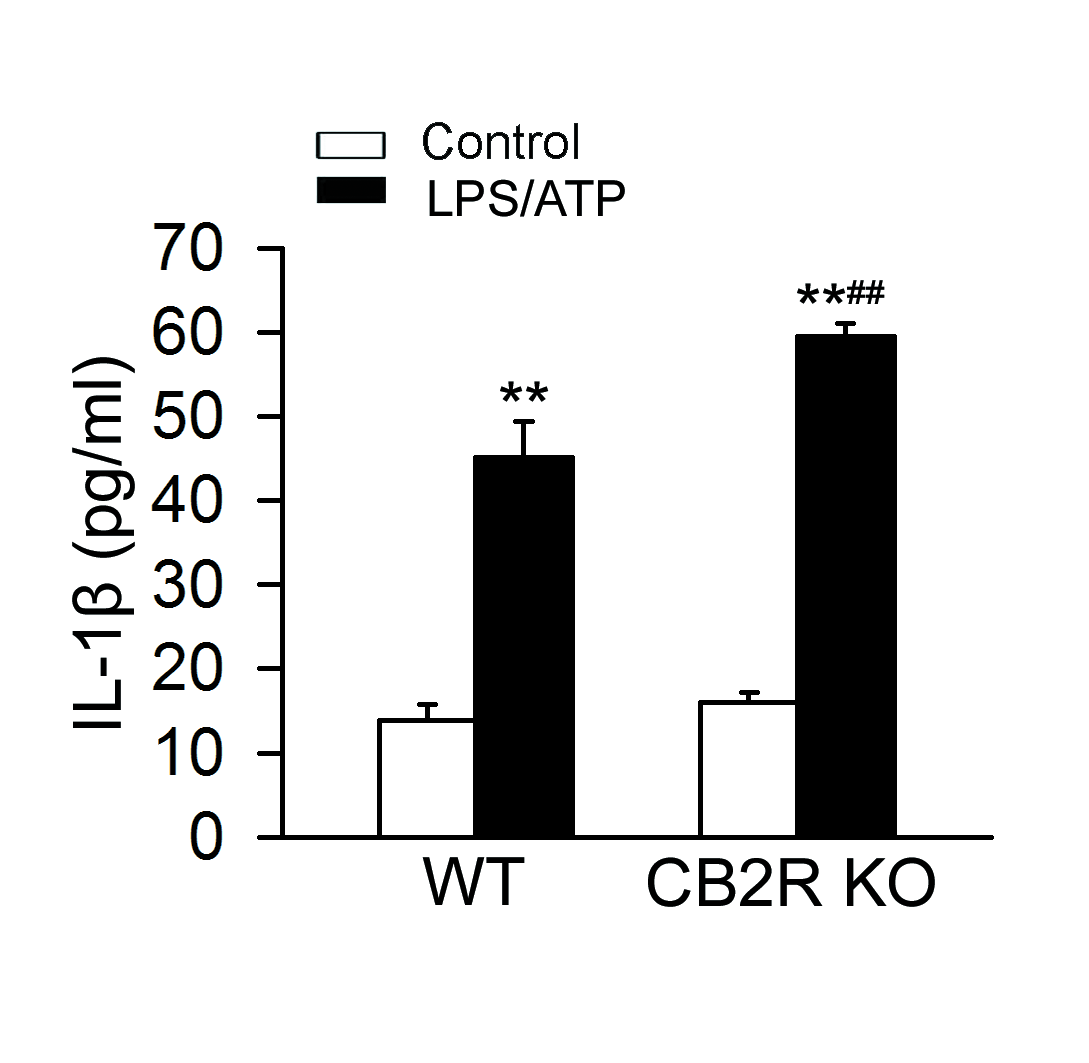

Supplement: S2 Fig — The level of IL-1β in supernatant was measured by ELISA. **P<0.01 vs. control; ##P<0.01 vs. WT. (TIF) [file pone.0155076.s002.tif]
